# Stronger Athlete Identity Is a Risk Factor for More Severe Depressive Symptoms After Musculoskeletal Injury in Pediatric Athletes: a Systematic Review

**DOI:** 10.1007/s12178-023-09828-0

**Published:** 2023-03-30

**Authors:** Anna L. Park, Kira Furie, Stephanie E. Wong

**Affiliations:** 1 University of California San Francisco School of Medicine, San Francisco, CA 94143 USA; 2 Department of Orthopaedic Surgery, University of California San Francisco, San Francisco, CA 94143 USA

**Keywords:** Mental health, Athlete identity, Pediatric, Musculoskeletal injury

## Abstract

**Purpose of Review:**

Treatment for musculoskeletal sports injuries often neglects the psychological components of health and recovery. Pediatric patients require particular consideration of their psychosocial and cognitive development. This systematic review investigates the effects of musculoskeletal injury on mental health in pediatric athletes.

**Recent Findings:**

Athlete identity may increase in adolescence and is associated with worse mental health post-injury. Psychological models suggest loss of identity, uncertainty, and fear mediate the association between injury and symptoms of anxiety, depression, post-traumatic stress disorder, and obsessive–compulsive disorder. Fear, identity, and uncertainty also influence return to sport.

**Summary:**

In the reviewed literature, there were 19 psychological screening tools and 8 different physical health measures with various adaptations to athlete developmental level. In pediatric patients, no interventions were studied to reduce the psychosocial impacts of injury. Musculoskeletal injury is associated with worse mental health in pediatric athletes, and stronger athlete identity is a risk factor for the development of depressive symptoms. Psychological interventions that reduce uncertainty and address fear may help mitigate these risks. More research is needed on screening and interventions to improve mental health post-injury.

## Introduction

Treatment for musculoskeletal injuries often focuses on physical and functional outcomes, neglecting the psychological components of health and recovery. However, psychological responses to injury including cognitive, emotional, and behavioral aspects are known to be important factors in determining outcomes post-injury [1••]. Athletes may respond to injury differently than their non-athlete counterparts; for example, they might be at higher risk of self-medication post-injury as part of coping [1••, 2••]. For this reason, the American Medical Society for Sports Medicine states that there is “a clear need for depression and anxiety screening, especially after an athlete has experienced a challenging illness or injury” [1••]. Pediatric athletes have special considerations regarding mental health including parental involvement and psychological developmental status of the athlete. For example, identity formation varies with age and may be affected differently at different ages [3•]. Therefore, it is important to investigate the pediatric-specific considerations for mental health post sports injury. Despite these calls for more robust research and guidelines, there is no clear understanding of how musculoskeletal injury affects mental health and no clear guidelines for treating mental health post-injury.

Injuries sustained by athletes can play a role in the development of psychiatric disorders. Emotional responses to injury that do not resolve or that worsen over time can be associated with higher rates of generalized anxiety disorder (GAD) [1••]. Injured athletes may also be at higher risk of developing depression, post-traumatic stress disorder, or obsessive–compulsive disorder. Furthermore, mental health disorders may present differently in athletes than their non-athlete counterparts. For example, “lack of focus” for an athlete may be a sign of cognitive functional impairment resulting from depression [1••].

The primary purpose of this systematic review is to investigate the effects of musculoskeletal injury on mental health in pediatric athletes. Secondary outcomes include reviewing the measures used to assess mental health post-injury and any interventions directed at improved mental health post-injury.

## Methods

### Search Strategy

We searched PubMed and PsycNet databases using ((pediatric OR adolescent OR child) AND (athlete OR sports OR athletic) AND (mental health) AND (injury)), which yielded 627 unique articles. Two authors (AP, KF) screened the titles and abstracts, and questions were resolved by discussion between them.

### Inclusion/Exclusion Criteria

We included articles that investigated mental health in pediatric athletes after musculoskeletal injury. Articles were excluded if they investigated the effects of mental health on subsequent injury risk, focused on concussion/traumatic brain injury, did not mention athletes or sport injury, or did not study pediatric populations. Non-peer-reviewed articles, letters to the editor, and books were excluded.

### Data Extraction and Synthesis

The measures used to assess mental and physical health were recorded, and key themes and theories were extracted from the included studies.

## Results

### Search Results

**Fig. 1:**
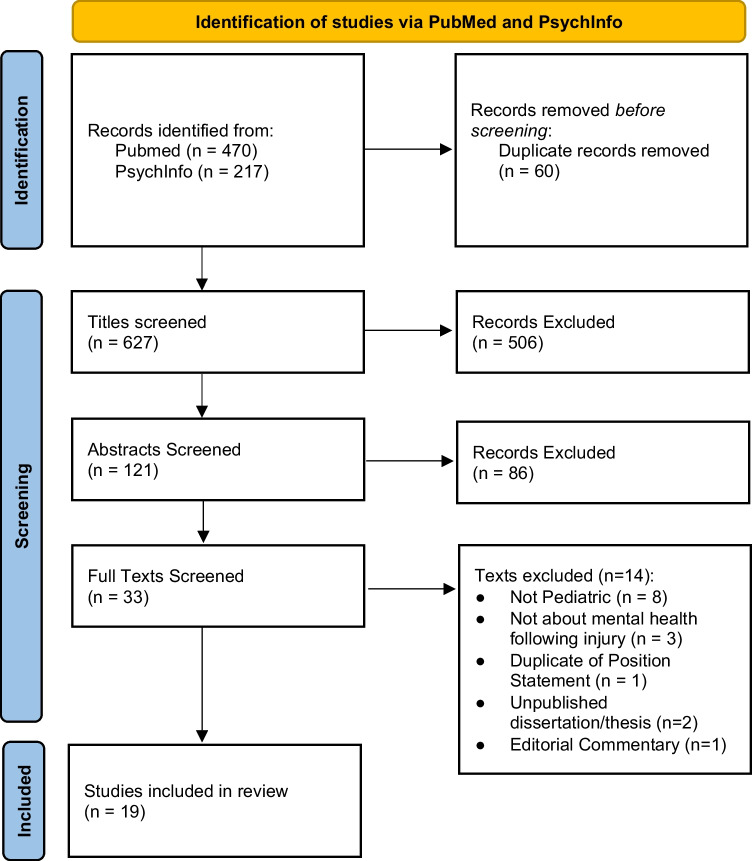
PRISMA diagram: title, abstract, and full-text review process yielded 19 articles for inclusion in the review

After title and abstract screening using the inclusion criteria, 33 articles were selected for full-text review. Further, 14 articles were excluded during the full-text review based on relevance, population studied, and type of publication (Fig. 1).

The 19 included articles consisted of 12 observational studies, 4 of which utilized interview-based methodology. There were 6 review articles which focused on themes including athlete identity, psychological stress related to injury, health-related quality of life, depression, and psychosocial impacts of injury in athletes. The remaining article was the American Medical Society for Sports Medicine 2020 position statement on mental health issues in athletes.

### Pediatric-Specific Athlete Mental Health

Two considerations that are particularly important for understanding mental health in pediatric populations are (1) the developmental stage of the athlete and (2) the parent/guardian–child relationship. Different developmental stages may predispose athletes to different types of mental health issues post-musculoskeletal injury. For example, athletes ages 15 to 21 may be more likely to have symptoms of post-traumatic stress disorder after injury than athletes under age 14 [3*•*]. Other studies suggest that pediatric athletes may have more difficulty coping with identity transition caused by injury when compared to adult athletes due to the large role that sports play in their lives [4*•*]. Additionally, managing parent/guardian anxiety may be an important part of promoting mental health post-injury in adolescent athletes [5].

### General Models for Mental Health After Musculoskeletal Injury

Models for mental health in athletes post-injury approach the topic from a variety of standpoints, drawing on literature relating to identity theory, psychiatric diagnosis, quality of life, and developmental stages. Generally, sports participation has been shown to have benefits on mental health, with athletes having lower risk of depressive symptoms than their non-athlete counterparts [2*••*, 3*•*, 5]. However, athletes may also have increased risk for major depressive episodes, which may be due to the demands of performance [3*•*, 5]. Injury is one of the most commonly cited events for development of mental health issues in athletes.

Injury is thought to affect mental health via multiple mechanisms. Athletic identity, self-esteem, and total mood disturbance are important factors affecting mental health post-injury [6]. An interview-based study identified 5 themes associated with quality of life after injury, including: relationships, uncertainty or fear, mood, stress and pressure, and energy [7]. The relationship between injury and these dimensions of psychological health suggests that health-related quality of life is an important measure for the recovery process in sports-related injury [7, 8].

Jasser et al. (2022) suggests that the emotional stages that athletes go through after injury may be similar to the process of grieving other losses [5]. These effects may be mediated by the patient’s athlete identity. Athlete identity (AI) is a measure of the strength and exclusivity with which a person identifies as an athlete [2*••*, 4*•*, 9*••*]. Some studies suggest that AI may increase during adolescence compared to later in training and that higher AI can protect against burnout and promote adherence to rehabilitation [2*••*, 9*••*]. However, higher AI has also been associated with increased risk of depression post-injury [9*••*]. One study suggested that younger patients may have more difficulty coping with injury due to the threat to their AI [4*•*].

Studies examining specific psychiatric conditions highlight that the conditions may present differently in athletes than in non-athlete populations [1*••*]. Among the articles included in this review, depression is the most studied psychiatric diagnosis after musculoskeletal injury. Risk factors for depressive symptoms post-injury include female gender, worse injury severity, high athletic identity, and lack of positive stress (stress that is beneficial, producing emotions such as excitement) [3*•*]. By contrast, multisport exposure, “mental toughness,” and resiliency may have a protective role for athlete mental health post-injury [1*••*, 5].

One study found that there is a common time course for depressive symptoms post-injury, where depressive symptoms are the highest immediately after injury and then decrease over time. These symptoms can be measured using the Centers for Epidemiological Studies Depression (CES-D) scale, which has a high sensitivity for detecting depression 1 week post-injury but may over-diagnose major depressive disorder later on [10]. Sports injury has also been associated with post-traumatic stress disorder in adolescents [3*•*]; however, none of the observational studies specifically examined diagnosis of PTSD in pediatric patients after injury.

### Observational Study Findings

Observational studies on mental health in pediatric athletes post-musculoskeletal injury focus on knee injuries. Anterior cruciate ligament (ACL) injuries are the most studied injury, while some studies also investigated meniscal tears, collateral ligament sprains, patellar instability, anterior knee pain, and femoral osteochondral lesions [11, 12]. Generally, knee injuries have been found to negatively impact physical and emotional health in athletes. McGuine et al. found that patients with less serious injuries (e.g., anterior knee pain) had similar health-related quality of life scores compared to those with more serious (e.g., ACL) injuries. The patients with less serious injuries had lower scores in social functioning, role physical, and bodily pain [11].

Research on mental health after ACL reconstruction (ACLR) highlights the importance of measuring mental health post-ACLR, specifically in pediatric populations. One study demonstrated a correlation between the Pediatric International Knee Documentation Committee (Pedi-IKDC) score and quality of life in social/emotional domains. Worse symptoms reported on Pedi-IKDC were associated with lower social and emotional quality of life in nonoperatively treated, preoperative, and post-operative pediatric ACL patients. In contrast, similar data from studies in adults does not demonstrate a correlation between IKDC and social/emotional quality of life [4*•*].

Studies on the markers of rehabilitation progress suggest that physical measures alone are insufficient to determine if an athlete is ready to return to sport. An interview-based study with high school student athletes found that there are significant psychological barriers to rehabilitation including fear of reinjury and uncertainty about readiness to return to play, warranting other measures in addition to the physical markers to evaluate progress post-ACLR [13*•*]. Important psychological factors during recovery include the type of support received from parents, coaches, and teammates; attention from physical therapists and surgeons; and the patient’s personal mental journey. For instance, overly sympathetic support or caution from others may negatively impact recovery or reduce likelihood of return to sport. Similarly, insufficient attention from physical therapists or the surgeon, specifically in relation to the patient’s goals, may negatively impact the patient’s recovery experience [13*•*]. Addressing psychological recovery after ACL injury, including the need to accept risk and face fear of reinjury, is essential to overall recovery and clinical outcomes [2*••*, 14].

Comparison between athletes in different types of sports found that patient-reported outcomes for mental health were similar for high school and collegiate injured in a variety of sport types, including soccer, football, skiing, baseball, lacrosse, and tennis [15]. However, other research suggests that factors such as team versus individual sport may affect athlete mental health. For example, athletes who develop social anxiety may have more challenges participating in team sports, such as basketball, than they would participating in individual sports or sports with fewer spectators such as distance running [5].

One way to approach tailoring care to individual patients is to evaluate motives for sports participation. Motives such as achievement, fun, and well-being have been shown to predict worse pain and functioning (as measured on KOOS) at 2 years post-injury when compared to the motives of health or social integration. These findings were not, however, correlated with worse mental component scores on the Short-Form-36 (SF-36), a quality of life scale [16].

One study investigated the potential for ACL injury, surgery, and recovery to promote adversarial growth (positive psychological change in response to adverse events). Their approach suggests potential positive effects of injury on long-term mental health. However, the study found that these injury events did not seem to promote adversarial growth [17].

### Return to Sport

Return to sport (RTS) was a focus of the pediatric literature on mental health post-injury. In patients who underwent ACLR, those who did not RTS were shown to have decreased health-related quality of life when compared to those who did or to healthy controls [18*•*]. Factors correlated with increased likelihood of return to sport include confidence in personal ability, support at all levels, enhanced physical ability, and active strategies involving cognitive awareness of stressors with conscious attempts to reduce negative outcomes such as social isolation [19]. While some reasons for not returning to sport were found to be external to the injury (e.g., graduation from high school) [12], others were related to physical and mental aspects of the recovery process.

Interactions with the medical system may also positively affect return to play after ACLR, specifically those that promote learning, trusting providers, personalization of care, and socialization [13*•*]. Learning about the recovery process can help reduce uncertainty, and more knowledge is associated with maintenance of a healthy mindset [1*••*, 13*•*]. Trusting relationships with physical therapists or athletic trainers are also important to RTS. At the high school level, school athletic trainers have been found to be important for athlete emotional stability post-injury [13*•*]. Personalized rehabilitation processes incorporating sport-specific training are also important promoters of RTS. Adding sport-specific exercises and guidance on the transition from rehabilitation back into normal training encourages RTS. Finally, injuries can be isolating, especially for adolescent athletes whose social networks revolve around sport. Socialization in the rehabilitation setting and finding ways to keep athletes involved in their sport can also help athletes build a social support system, promoting RTS [13*•*].

One semi-structured interview study investigated the psychological aspects of RTS after injury. They characterized the mental process of RTS in three main themes: driving reasons for RTS, preparation of body and mind, and risk acceptance. Driving reasons to RTS include athletic identity, mental toughness, and commitment to the self, sport, or team. Preparation of body and mind included connectedness, physical rehabilitation, and cognitive skills and planning. Finally, risk acceptance was facilitated by graded exposure in transition to full sport, which allowed athletes to approach chaos and confusion with confidence [14]. Overarching narratives for injury processes may also be important for return to sport. There is some evidence that viewing injury as “overcoming adversity” promotes return to sport [13*•*].

Barriers to RTS include physical limitations, social factors, fear, and uncertainty in both the rehabilitation progress and transition back to sport. Physical barriers such as fatigue, stiffness, pain, discomfort, and limited strength and mobility negatively impact RTS [13*•*, 19]. Social factors such as discouraging interpersonal comparison to other injured athletes during physical therapy challenge RTS. Outside the clinic, external support, identity challenge, and role adjustment also impede RTS [13*•*, 19]. One study suggests that female athletes generally receive less support at all levels including from friends and family members, contributing to female athletes being less likely to RTS after injury [19]. Uncertainties about progress in rehabilitation, about how physical therapy exercises relate to sporting activities, and about how to transition back into full sport are all barriers athletes faced in returning to sports [13*•*].

Fear is an important psychological factor affecting return to sport and outcomes post-musculoskeletal injury [12, 13*•*, 18*•*]. Athletes who did not return to sport after ACLR were found to have higher fear-avoidance beliefs when compared to those who did return. However, the athletes in this study that did successfully return to sport had higher fear-avoidance beliefs than healthy controls [18*•*].

### Measures for Mental Health and Injury

There were 19 unique psychological measures used in the studies or suggested as screening tools for mental health complications after injury in pediatric patients. Two measures assess athletic identity, 5 assess health-related quality of life, 5 assess mood/depressive symptoms, 3 assess anxiety and post-traumatic stress symptoms, and 4 assess fear-avoidance beliefs and behaviors (see Table 1). Additionally, Jasser et al. (2022) provide a list of sport-related psychosocial screening questions that may be used to assess for underlying mental health concerns in pediatric athletes [5].

**Table 1:** Psychological measures used to assess mental health in pediatric athletes

Measure	Type	Subscales	Used to measure
Athletic identity			
Athletic Identity Measurement Scale (AIMS) [9*••*]	7-item questionnaire on a 7-point Likert scale from “strongly agree” to “strongly disagree”	Social identity, exclusivity, negative affectivity	Measures athletic identity as a cognitive structure and social role. Validated in youth student samples
Anderson’s Athletic Identity Questionnaire (AIQ) for Adolescents [9*••*]	21-item “yes/no” questionnaire	Appearance, importance of exercise/sport/physical activity to the self, perceived competence, encouragement from others participating	Measures athlete identity across four dimensions of self-knowledge. Validated in adolescents
Quality of life			
Child Health Questionnaire (CHQ) CHQ-CF87 version [4*•*]	87-item self-report, available in multiple languages	Overall physical, psychosocial	Assesses health-related quality of life in people age 5 to 18. There are both parent- and patient-reported versions of varying lengths of the CHQ [20]
Disablement in the Physically Active Scale (DPA) [7] and Modified Disablement in the Physically Active Scale (mDPA) [18*•*]	16-item questionnaire on a 5-point Likert scale	Physical components, mental components	Assesses health-related quality of life
Pediatric Quality of Life (PedsQL) [8]	23-item questionnaire	4 domains of health-related quality of life: physical, emotional, social, school functioning	Measure of health-related quality of life
Short-Form 36 (SF-36) [16]	36-item questionnaire	Physical health (physical functioning, role physical, bodily pain, general health), mental health (vitality, social functioning, role emotional, mental health)	Global health-related quality of life survey
Short-Form 12 (SF-12) [11, 12, 15]	12-item questionnaire. Available in more than 50 languages	Physical health (physical functioning, role physical, bodily pain, general health), mental health (vitality, social functioning, role emotional, mental health)	Global health-related quality of life survey. Validated shortened version of SF-36, and used in patients as young as 14
Depression/mood			
Beck Depression Inventory (BDI) [1*••*]	21-item questionnaire on a 4-point Likert scale	Cognitive, somatic-affective [21]	Screening tool for depression. Not validated in athletes
Centers for Epidemiological Studies Depression (CES-D) [1*••*, 10]	20-item questionnaire on a 4-point Likert scale from “Rarely or none of the time (less than 1 day)” to “Most or all of the time (5–7 days)”	Cognitive, affective, behavioral, somatic	Self-report survey of depressive symptoms. Reliably associated with major depressive disorder, and used in children
Hamilton Rating Scale for Depression (SIGH-D) [10]	Structured interview guideline and subscale for the Hamilton Rating Scale. Available in German, Italian, Polish, and Chinese	Depression	Clinical interview-based assessment for mood and anxiety disorders
Patient Health Questionnaire 2 and 9 (PHQ2 and PHQ9) [1*••*]	2- or 9-item questionnaire on a 4-point Likert scale	DSM-5 criteria for depression	Screening tool for depression. Not validated in athletes
Profile of Mood States (POMS) [10, 17, 22]	37-item shortened POMS questionnaire condensed from the original 65-item POMS. There is also a 6-item version called the Brief Assessment of Mood (BAM)	Tension, anger, depression, confusion, fatigue, vigor	Measures mood states including depressed mood
Anxiety/stress disorders			
Generalized Anxiety Disorder – 7 (GAD-7) [1*••*]	7-item questionnaire on a 4-point Likert scale	DSM-5 criteria for generalized anxiety disorder	Screening tool for anxiety disorders
Horowitz Impact of Event Scale-Revised (IES-R) [9*••*]	22-item questionnaire on a 5-point Likert scale from “not at all” to “extremely”	Intrusion, avoidance, hyperarousal [23]	Measures symptoms of PTSD. Not established in orthopedic literature for assessing trauma [9]
Post-traumatic Growth Inventory (PTGI) [17]	21-item questionnaire on a 6-point Likert scale.	New possibilities, relating to others, personal strength, spiritual change, appreciation of life	Measures adversarial growth after traumatic event
Fear avoidance			
Anterior Cruciate Ligament – Return to Sport Index (ACL-RSI) [14]	12-item “yes/no” questionnaire.	Emotions, confidence in performance, risk appraisal	Measures the cognitive side of return to sport after ACL injury
Athlete Fear Avoidance Questionnaire (AFAQ) [13*•*]	10-item questionnaire on a 5-point Likert scale	No subscales	Measures injury-related fear avoidance in athletes [24]
Fear Avoidance Belief Questionnaire (FABQ) for Athletes [18*•*]	16-item questionnaire on a 7-point Likert scale	Physical activity, sport	Assesses fear-avoidance beliefs/behaviors
Tampa Scale of Kinesiophobia-11 (TSK-11) [13*•*]	11-item questionnaire. Other versions exist with variable numbers of question	Somatic focus, activity avoidance	Measures fear of movement/reinjury validated in chronic pain patients [25]

Eight different physical health measures are used in the studies to measure functioning post-injury: International Knee Documentation Committee (IKDC) [11, 12, 14, 15], Pediatric International Knee Documentation Committee (Pedi-IKDC) [4*•*], Lysholm-Tegner Scales [12–15], Western Ontario and McMaster Universities Osteoarthritis (WOMAC) [12], Marx Activity Scale [12, 15], Knee Injury and Osteoarthritis Outcome Score (KOOS) [13*•*, 16], Knee Outcomes Survey – Sports Activities Scale (KOS-SAS) [17], and a numerical rating scale for pain [17].

## Discussion

### Effects of Injury on Pediatric Athlete Mental Health

Our findings from the reviewed literature highlight the importance of understanding pediatric-specific and athlete-specific factors affecting mental health after musculoskeletal injury. Adolescent athletes may be specifically vulnerable to worse mental health outcomes post-injury due to the natural changes in identity formation associated with their developmental stage [13*•*]. The process of identity formation during adolescence can affect ability to cope with further identity challenge caused by injury, which may more frequently lead to development of depression or PTSD for adolescents when compared to young children or adults [3*•*, 13*•*]. One study in adult patients found that athletes with a history of injury had a greater physiological stress response measured via skin conductance to watching videos of orthopedic injuries than those with no history of injury [22]. Similar studies in pediatric patients could help elucidate the underlying relationships between age, injury, and psychiatric disorders.

Mental health outcomes after injury may also be affected by social dynamics, parental involvement, and sports specialization [2*••*, 5]. Given that recent trends towards early sport specialization in children are thought to contribute to increased risk of injury and burnout among young athletes [26], further research in pediatric populations is needed to better understand how injury interacts with other risk factors to predispose athletes to worse mental health post-injury.

Overall, the literature focused on mental health challenges for athletes post-injury. The one study that looked at the potential positive impact of injury on mental health did not find that injury promoted adversarial growth [17]. However, it is still possible that injury and rehabilitation could promote other positive aspects of mental health such as resiliency. Future research in this area should be aware of these potential psychological impacts of injury.

The rest of the reviewed studies focused on the relationship between injury and poor mental health, connecting injury with development of depression, post-traumatic stress disorder, and anxiety disorders. A variety of risk factors may mediate these relationships including age, personality, type of sport played, and support systems. The nature of these relationships remains unclear. Furthermore, common mental health disorders such as depression or anxiety may present differently in athletes than non-athletes [1*••*]. For these reasons, further research and modeling are necessary for understanding how musculoskeletal injury affects mental health.

### Interventions to Promote Better Mental Health Post-Injury

There were no interventional studies to improve mental health in pediatric athletes after injury. Despite the lack of interventional trials, there were a number of suggestions for interventions in the literature, which fall into four categories: (1) reducing uncertainty, (2) promoting support, (3) maintaining connection to sport, and (4) connection to psychological services. In order to reduce uncertainty, healthcare providers may educate the athlete on the healing process and what to expect, including a clear transition process and timeline for reintroduction to full sport and training if that is the patient’s goal. Proper social support for pediatric athletes can include parents, coaches, athletic trainers, teammates, friends, and others. Specifically educating parents/guardians about how their own anxiety may affect their child could reduce the negative effects of injury on mental health. Continued integration in their sport, for example, by finding ways for athletes to participate at practice, or designing sport-specific exercises and training programs can help promote positive mental health post-injury [2*••*]. Finally, integrating psychologists into the care team for injured athletes can improve access to beneficial interventions [2*••*, 5]. Psychologists may also be able to provide targeted evidence-based interventions such as cognitive behavioral therapy, family therapy, or motivational interviewing for pediatric patients who develop or are at risk of developing poor mental functioning after injury [2*••*, 5].

Return to sport is a factor related to mental health post-injury that is particularly important for pediatric populations. Psychological factors such as fear avoidance may affect return to sport, or conversely, return to sport may affect psychological factors important to mental health. In pediatric populations, negative mental health effects after injury may impact scholastic and social functioning, which could have a major impact on future health, functioning, and life goals. Furthermore, fear-avoidance behavior is associated with the development and maintenance of chronic pain in the pediatric fear-avoidance model of chronic pain [27]. Given the association between return to sport and lower fear-avoidance behavior, one’s ability to successfully return to sport may also potentially decrease the risk of development of chronic pain. Thus, managing mental health post-injury should be considered an important factor in promoting long-term health and wellness.

Our study has several limitations. Firstly, all the observational research was conducted on knee injuries, and mostly ACLR, limiting the external validity of the reviewed data. Secondly, the research is also done across a variety of sports, which makes drawing conclusions about the impact of injury on mental health difficult as different factors or effects may be more or less important for certain sports. Thirdly, many of the instruments for measuring psychological health and functioning are not validated specifically for athletes or in pediatric populations. Finally, there was no discussion of how social determinants of health may affect the ability to cope with injury, the ability to access mental healthcare, or recovery process generally.

## Conclusions

Overall, musculoskeletal injury in pediatric athletes is associated with poor mental health and functioning, which affects clinical outcomes as well as overall health and well-being of the athlete. Strong athlete identity is a risk factor for more depressive symptoms after injury.

While there were no interventional studies in the reviewed literature, recommended practices include reducing uncertainty, promoting support from medical team and family, maintaining the athlete’s connection to sport, and connecting the athlete to psychological services. Further research is needed to better understand this relationship and to test interventions to improve mental health post-injury.
